# Molecular Detection of Soil-Transmitted Helminths and Enteric Protozoa Infection in Children and Its Association with Household Water and Sanitation in Manhiça District, Southern Mozambique

**DOI:** 10.3390/pathogens10070838

**Published:** 2021-07-03

**Authors:** Berta Grau-Pujol, Inocencia Cuamba, Chenjerai Jairoce, Anelsio Cossa, Juliana Da Silva, Charfudin Sacoor, Carlota Dobaño, Augusto Nhabomba, Rojelio Mejia, Jose Muñoz

**Affiliations:** 1 Barcelona Institute for Global Health (ISGlobal), Hospital Clínic—University of Barcelona, 08036 Barcelona, Spain; carlota.dobano@isglobal.org (C.D.); jose.munoz@isglobal.org (J.M.); 2 Centro de Investigação em Saúde de Manhiça (CISM), Maputo 1929, Mozambique; inocencia.Cuamba@manhica.net (I.C.); chenjerai.Jairoce@manhica.net (C.J.); anelsio.cossa@manhica.net (A.C.); charfudin.Sacoor@manhica.net (C.S.); augusto.nhabomba@gmail.com (A.N.); 3 Mundo Sano Foundation, Buenos Aires 1535, Argentina; 4 Department of Medicine, Baylor College of Medicine, Houston, TX 77030, USA; lxi7@cdc.gov; 5 Department of Pediatrics, National School of Tropical Medicine, Baylor College of Medicine, Houston, TX 77030, USA; Rojelio.Mejia@bcm.edu

**Keywords:** Mozambique, protozoa, soil-transmitted helminths, *Giardia*, sub-Saharan Africa, intestinal parasites, PCR, water, sanitation, poverty

## Abstract

Intestinal parasite infections can have detrimental health consequences in children. In Mozambique, soil-transmitted helminth (STH) infections are controlled through mass drug administration since 2011, but no specific control program exists for enteric protozoa. This study evaluates STH and protozoan infections in children attending healthcare in Manhiça district, Southern Mozambique, and its association with water and sanitation conditions. We conducted a cross-sectional study in children between 2 and 10 years old in two health centers (n = 405). A stool sample and metadata were collected from each child. Samples were analyzed by multi-parallel real-time quantitative PCR (qPCR). We fitted logistic regression-adjusted models to assess the association between STH or protozoan infection with household water and sanitation use. Nineteen percent were infected with at least one STH and 77.5% with at least one enteric protozoon. qPCR detected 18.8% of participants with intestinal polyparasitism. Protected or unprotected water well use showed a higher risk for at least one protozoan infection in children (OR: 2.59, CI: 1.01–6.65, *p*-value = 0.010; OR: 5.21, CI: 1.56–17.46, *p*-value = 0.010, respectively) compared to household piped water. A high proportion of children had enteric protozoan infections. Well consumable water displayed high risk for that.

## 1. Introduction

Intestinal parasites are transmitted by the fecal-oral route or by skin penetration. Thus, they mostly affect deprived populations, and specifically children, with lack of hygiene, safe water, and improved sanitation access. Their infections can be asymptomatic or cause abdominal pain, diarrhea, malnutrition, anemia, and impaired development and growth [1,2,3]. 

Soil-transmitted helminths (STH) are intestinal parasites that affect approximately 1.5 billion people worldwide [2]. The World Health Organization (WHO) formally consider STH as roundworm (*Ascaris lumbricoides*), whipworm (*Trichuris trichiura*), and hookworm (*Necator americanus* and *Ancylostoma duodenale*). WHO set the goal to reduce moderate and high intensity STH infections to less than 2% in 96% of endemic countries by 2030 [4]. For that, the main STH control strategy is mass drug administration (MDA) with albendazole or mebendazole to populations at risk once a year if prevalence is over 20% and twice a year if it is over 50%. MDA-continued campaigns have substantially reduced STH infections in many countries [5,6,7]. Due to ongoing transmission, the WHO recommends improving water, sanitation, and hygiene (WASH) strategies to reduce STH reinfection, but it has been poorly accomplished [8].

Other intestinal parasites are not targeted by a specific control program neither can benefit from STH MDA [9]. *Strongyloides stercoralis* or enteric protozoa such as *Giardia lamblia*, *Entamoeba histolytica,* and *Cryptosporidium spp* are examples of those [10]. Indeed, global distribution and burden for these four parasites have been unrepresented for many years. For *S. stercoralis*, two recent studies estimated their burden (386 million people infected) [11,12]. Regarding enteric protozoa, the estimated global burden in 2010 was almost 3 million Disability Adjusted Life Years (DALYs) [13]. Nevertheless, they are estimations in response to missing regional and national data. Moreover, most surveillance studies have not used sensitive molecular diagnostic techniques [14,15].

Mozambique is a country in sub-Saharan Africa where 72% of the population have access to safe water and only 39% utilized improved sanitation in 2015 [16]. Occasional studies have reported STH and *S. stercoralis* infections in Mozambique for decades [17], but current published data are scarce. Concerning enteric pathogens, although they were described in children under 5 [18,19], a small number of studies examined enteric protozoa infections in those over 5 years old in Mozambique [20,21]. 

In this study, we aim to describe STH and enteric protozoa infection prevalence in children between 2 and 10 years old attending healthcare in Manhiça District, Southern Mozambique, using a highly sensitive diagnostic technique, multi-parallel real-time quantitative PCR (qPCR). In addition, we assessed the association between STH and protozoan infections with household’s water and sanitation facilities used. 

## 2. Results

### 2.1. Participant’s Characteristics

We recruited 405 children, 232 from Manhiça-sede and 173 from Ilha Josina health centers. Half (51.6%) were male and 52.1% were between 2 and 5 years old. The proportion of participants belonging to a poor socioeconomical status was 24.2%, 12.1% in Manhiça-sede, and 40.5% in Ilha Josina. Regarding household water source facilities, 46.4% of study participants were living in a household with piped water (76.3% in Manhiça-sede and 6.3% in Ilha Josina) and 17.3% were living in a household using unprotected well (3.4% in Manhiça-sede and 35.8% in Ilha Josina). Regarding household sanitation facilities, 63% of participants were living in a household using an improved latrine (51.7% in Manhiça-sede and 78% in Ilha Josina), followed by 15.3% who were using unimproved latrine (22.4% in Manhiça-sede and 5.8% in Ilha Josina), and 12.1% who were using a toilet connected to a septic tank (20.7% in Manhiça-sede and 0.6% in Ilha Josina). Six percent of participants did not have a latrine at home, 3.4% in Manhiça-sede and 9.8% in Ilha Josina. Eighteen participants were HIV positive (Table 1).

### 2.2. STH and Protozoan Infections

**Figure 1:**
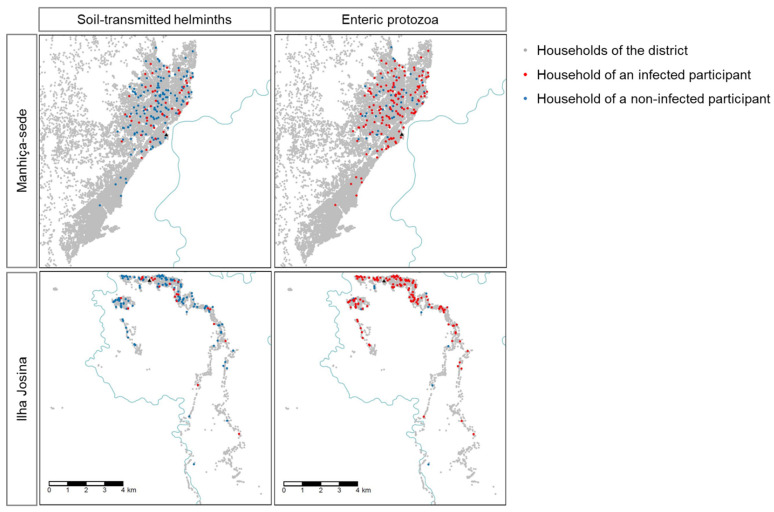
Location of participants infected with at least one soil-transmitted helminth (STH), including *Strongyloides stercoralis*, or one enteric protozoon. Incomati river is displayed in blue.

Almost a fifth of participants (19.3%) were infected with at least one STH (including *S. stercoralis*) detected by multiparallel qPCR, 22.8% of participants in Manhiça-sede, and 14.5% in Ilha Josina (Figure 1). *S. stercoralis* (8.6%) and *A. lumbricoides* (7.4%) were the most frequently detected, followed by *T. trichiura* (4%), *N. americanus* (3%), and *A. duodenale* (0.5%) (Table 2). A higher proportion of STH infected were observed in children older than 5 years old (24.7%) compared to 2–5 years old group (14.2%, *p*-value = 0.008). We did not observe any difference in the proportion of STH-infected children between HIV infected or not (16.7% and 19.5%, respectively, *p*-value = 1.000). 

Regarding protozoa, 77.5% of participants were infected by at least one enteric species, 70.7% of participants of Manhiça-sede and 86.7% in Ilha Josina (Figure 1). Multi-parallel qPCR detected 77.3% of analyzed samples *Giardia lamblia* positive, 5.7% *Cryptosporidium parvum/hominis* positive, and 0.7% *Entamoeba histolytica* positive (Table 2). We did not observe differences in the proportion of enteric protozoa-infected children between those 2–5 years old and those 5 to 10 years old (79.2% and 75.8%, respectively, *p*-value = 0.475), neither between HIV-infected or uninfected (72.2% and 77.9%, respectively, *p*-value = 0.566). 

### 2.3. Polyparasitism

**Figure 2:**
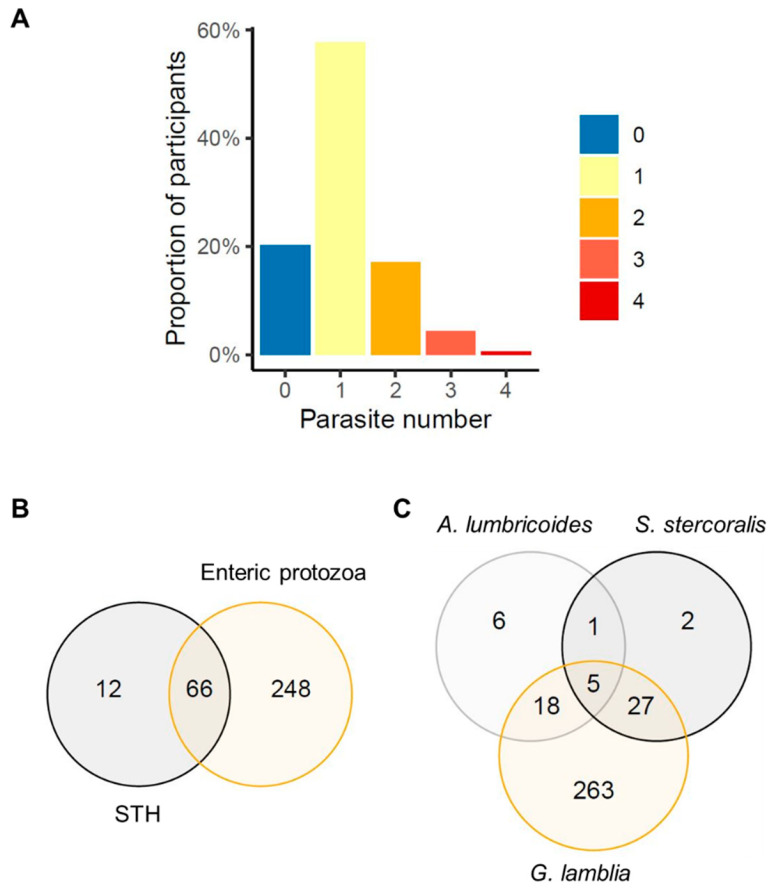
Participants’ polyparasitism, (**A**) proportion of participants per number of intestinal parasites, (**B**) number of participants with soil-transmitted helminth (STH), including *Strongyloides stercoralis,* and enteric protozoa coinfection, (**C**) number of participants coinfected with the most prevalent intestinal parasites in our study: *Ascaris lumbricoides*, *S. stercoralis* and *Giardia lamblia*.

A proportion of 18.8% of participants were infected by two or more intestinal parasites (either STH or enteric protozoa), 22.4% in Manhiça-sede and 13.9% in Ilha Josina. Sixteen percent of participants had a coinfection between at least one STH and at least one enteric protozoon. *G. lamblia* and *S. stercoralis* were the most common coinfection (7.9%), followed by *G. lamblia* and *A. lumbricoides* (5.7%). Moreover, three participants were infected by four different intestinal parasites and one participant was infected by five different intestinal parasites (Figure 2).

### 2.4. Association Between Household Water and Sanitation Facility Use and STH and Enteric Protozoa Infection

Children living in a household using protected water well (OR: 2.59, CI: 1.01–6.65, *p*-value = 0.010) or unprotected well (OR: 5.21, CI: 1.56–17.46, *p*-value = 0.010) displayed a higher odds ratio for infection with at least one protozoon compared to using piped water inside the household after adjusting for participant’s age, sex, household latrine type, socioeconomical score, and household distance to the health post. We did not observe any association between household water source and at least one STH, neither between sanitation facility used in the household and at least one STH or at least one protozoon infection (Table 3, Figures S2 and S3).

## 3. Discussion

During 2015−2016, we recruited and assessed STH and enteric protozoan infections in 405 children between 2 and 10 years old attending healthcare in Manhiça district, Southern Mozambique. Among them, *G. lamblia* infection was the most prevalent by far, 77.3%. This is the highest prevalence of giardiasis reported in Mozambique in the past decades. 

Certainly, this is a hospital-based cross-sectional study, thus, biases toward sick and symptomatic children. However, a cross-sectional study in Zambezia province, Mozambique, during 2017–2019 observed similar *Giardia* infection PCR detected prevalences in health centers and in schools in children between 3 and 14 years old (42% and 41.7%, respectively) [21]. Moreover, other studies in other Mozambican regions also showed *Giardia* infection among the most prevalent intestinal parasites. However, their prevalence assessed were lower than in our study. They used techniques with different sensitivity such as ELISA [22,23,24], and direct microscopy or Ritchie’s concentration technique [25]. But, in addition, environmental and socioeconomical conditions can be dissimilar inside the country and prompt different patterns of *Giardia* contamination [26]. 

The other enteric protozoa surveilled, *Cryptosporidium spp*. and *E. histolytica*, were less prevalent in children attending healthcare in Manhiça district. Regarding *Cryptosporidium spp*, other hospital-based studies in Mozambique also detected prevalence below 6% by microscopy or PCR [21,24]. Nonetheless, *Cryptosporidium spp*. was identified as one of the main causes of moderate to severe diarrhea in children under 5 years old in Manhiça district [19]. Concerning *E. histolytica*, we only observed 0.7% of children infected. The Global Enteric Multi-center Study (GEMS) also detected only 0.4% infection in hospitalized children for diarrhea in Manhiça district in 2007 [27]. Meanwhile, *E. histolytica* cysts antigens were detected by immunoassays and *E. hystolitica/dispar* was identified by microscopy in Nampula province [22,24], but other studies using molecular diagnostics did not detect *E. histolytica* in Zambezia province [18,20]. Furthermore, most cross-sectional studies on enteric protozoa infection in the country are in children younger than 5 years old [19,27]. In our study, including children older than 5 years old did not seem to determine prevalence of at least one enteric protozoon infection, since children younger than 5 years and older than 5 years old showed similar enteric protozoa infection prevalence.

A proportion of 19.3% of children were infected by at least one STH, including *S. stercoralis*. Regarding those species formally considered STH by WHO, *A. lumbricoides* was the most prevalent (7.4%), followed by *T. trichiura* (4%), *N. americanus* (3%), and *A. duodenale* (0.5%). In fact, *S. stercoralis* infection was higher (8.6%) than any formal STH species. Regarding age, it is understood that older children are not only breastfeeding, they freely move around barefoot and, thus, they can accidentally ingest contaminated food or soil [28]. Our results support that a higher proportion of STH-infected participants were older than 5 years old. This STH infection results are very valuable since last published data on STH infection and on *S. stercoralis* infection in southern Mozambique were from 2007 and 1995, respectively; before national MDA implementation [17,29,30]. Indeed, we observed less than half the prevalence identified before MDA implementation even though we used a more sensitive diagnostic technique [28,29]. Thus, MDA could have been effective in reducing STH infection in this region. 

Nineteen per cent of participants were infected by more than one intestinal parasite and 16% were infected by a STH and an enteric protozoan. The most common detected co-infections were *G. lamblia* with *S. stercoralis* and *G. lamblia* with *A. lumbricoides*. Clearly, intestinal parasites infections can have detrimental consequences in children, who are in a physical and intellectual development process. But the synergic impact of coinfection with either multiple STH and/or protozoa in children is still understudied and not well understood [26]. In addition, this study was conducted in an area with high HIV infection in adults with mother-to-child transmission below 5% [31,32]. While we did not find any statistical difference on intestinal parasite infection regarding HIV infection in our study, Acácio et al. (2018) reported that HIV infection increased the risk of moderate to severe diarrhea caused by *Cryptosporidium spp.* in children under 5 years old in Manhiça district [33]. 

In this study, we observed a higher odds ratio for enteric protozoa infection when children lived in a household using protected or unprotected water well. This goes in line with other studies [20,34]. The three enteric protozoa detected were foodborne and waterborne, they are typically transmitted by ingesting cysts persisting in water. Thus, lack of water quality and quantity and lack of hygiene will facilitate their transmission. Hence, well water could be contaminated from nearby latrines, domestic animals, or other contaminated users. Quantity of water acquired from wells could be insufficient since they involve physical fitness and time for water collection and; these conditions contribute to lack of hygiene [35]. However, Bauhofer et al.’s (2020) study in Mozambique found no association between water use and enteric protozoan infection [22]. Moreover, other studies focusing on water treatment did not find any association either, since cysts can be chlorination resistant [24,36,37]. On the other hand, we did not find any association between sanitation and protozoa infection. Thus, water source might be the main *Giardia* transmission source in this area.

Concerning STH, we did not observe any association with water and sanitation use. Other studies point that other unaccounted factors could be playing a role, such as soil conditions, household density, latrine sharing, latrine cleanliness, washing hands with soap, or school WASH conditions [26,29]. In addition, zoonotic infections could be also occurring [34]. 

This study has some limitations. First, we are reporting healthcare children prevalence, and community prevalence could differ. Second, we collected one stool sample per participant. Eggs, larvae, and cysts release in feces is intermittent, thus, prevalence could still be underestimated [14]. Third, not all participants were tested for all parasites. DNA extraction and multi-parallel PCR reagents were shipped from Houston, Texas, and CISM lab personnel were trained to perform multi-parallel qPCR in Mozambique. Damage of sensitive reagents due to suboptimal international shipment handling prevented us from performing qPCR in all intended samples. Fourth, qPCR has been discussed to detect other parasite specimens than the eggs or the cysts. Thus, this could affect qPCR specificity [38]. Moreover, this study was completed prior to the universal use of an exogenous internal control. Although and internal control was absent from this study, the DNA extraction method used has previously shown high fidelity with low inhibition. Fifth, hygiene, environmental, school and water and sanitation infrastructure operability and accessibility data were not available. This may have failed to capture WASH-related factors that could also be contributing to intestinal parasites transmission. 

However, the main strength of this study is that all intestinal parasites were identified by a highly sensitive and specific diagnostic technique on site—multi-parallel qPCR. This technique is less sensitive to stool texture and freshness than direct microscopy or Kato-Katz and allowed us to differentiate pathogenic from nonpathogenic parasites (e.g., *E. histolytica*, E. dispar) [39]. 

STH are mainly controlled through MDA with anthelmintics to population at risk. Nevertheless, MDA does not prevent from reinfection and researchers are uncovering anthelmintic resistance emergence [40]. The official inclusion of WASH improvements in STH national control programs could contribute to reduce STH reinfection and to achieve STH elimination as a public health problem [41]. In addition, WASH improvement is a long-term strategy that could prevent from other WASH related pathogens, such as *S. stercoralis* or enteric protozoa and, furthermore, it would enhance population living conditions.

## 4. Materials and Methods

### 4.1. Study Area

The study was carried out in Manhiça district, in Southern Mozambique, during August 2015 and December 2016 in the context of EcoHeMa study. Recruitment was conducted in two areas: Manhiça-sede, a peri-urban area of 78,479 inhabitants, and Ilha Josina, a rural area of 8288 inhabitants. (Figure S1) The climate is subtropical with a warm and rainy season (November to April) and a cool and dry season (June to October). The average annual temperatures oscillate from 22 °C to 24 °C and the average annual precipitation from 600 mm to 1000 mm.

Since 1996, the Manhiça Health Research Centre (Centro de Investigação em Saúde de Manhiça, CISM) runs a Demographic Surveillance System (DSS) covering the entire district (201,845 inhabitants). Every district household is geolocalized and every resident has a permanent identification number (PermID) issued by the DSS. The demographic trends in Manhiça District have been described in detail elsewhere [42].

Manhiça district followed expansive population pyramid distribution [42]. Houses are simple, with walls typically made of cane and concrete blocks, covered by zinc plates. The main occupations are farming, petty trading, and employment on a large sugar cane estate. The overall community prevalence of HIV/AIDS in the district was 40% [42].

### 4.2. Study Population

This study was a nested study inside EcoHeMa study, which aims to determine the prevalence of co-infection between *P. falciparum* malaria and helminthiasis at the hospital level. Recruitment was conducted in Manhiça-sede District Hospital and Ilha Josina Health center. (Figure S1) We included children aged between 2 and 10 years old, resident in the DSS area, presenting themselves at the study health centers and that their guardians gave informed consent. We excluded all children having received anthelmintics and/or antimalarial in the last 30 days.

In the absence of updated data, a conservative STH prevalence of 50% will ensure a sample size enough to achieve a precision of 5% in the estimation of the 95% CIs regardless of the true prevalence. Thus, an estimated sample size of 384 participants allowed us to answer our question. We enrolled 405 children DSS resident between 2–10 years old visiting a study health center, including those that were finally hospital admitted.

### 4.3. Study Design

**Figure 3:**
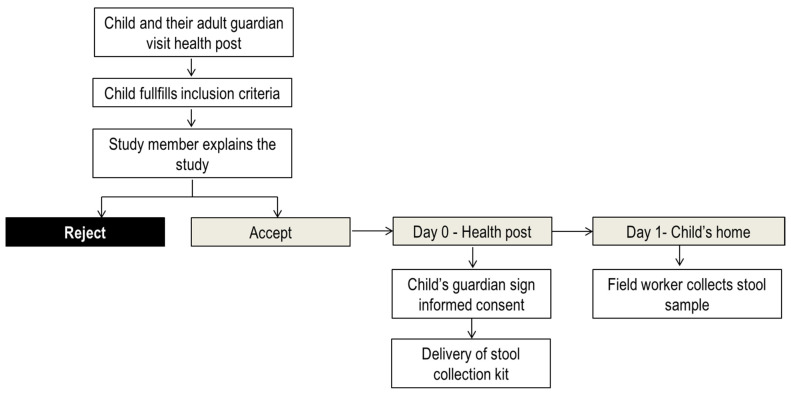
Stool sample collection flow diagram.

It was a cross-sectional study. After child adult guardian inform consent, a clinical study member explained sample collection methodology to the participants and provided a feces collection kit. The kit consisted of one sterile flask of 50 mL for stool sampling and three pairs of surgical gloves for protection. The field worker collected the stool sample the following morning after recruitment at participants’ households. In case stool samples could not be delivered that day, the field worker came back to the household until five consecutive days. Samples of hospital admitted participants were collected at the hospital (Figure 3).

### 4.4. Sample Examination

After collection stool samples were refrigerated at +/−4 °C in cool boxes containing cold packs and transported to the laboratory within 8 h. Stool samples were stored at −80 °C and processed in CISM laboratory. Participant samples were thawed and analyzed for molecular diagnosis by a multi-parallel quantitative real-time qPCR technique [39] in duplicate. DNA was extracted from 50 mg stool by using the MP FastDNA for Soil Kit (MP Biochemicals, Solon, OH, USA) according to manufacturer’s instructions [43]. Briefly, positives were determined based on the dynamic range of a standard set of specific parasite sequences. A sample was considered negative if the cycle threshold was (Ct) > 38 based on the limit of detection to parasite DNA sequences. Species-specific primers and FAM-labeled MGB probes were selected (Applied Biosystems, Forest City, CA, USA) for each of the 8 parasites (Table S1). Primer/probes were designed using Primer Express Software v3.0.1 (Applied Biosystems). Every reaction was performed in a total volume of 7 µL containing 3.5 µL Taqman™ fast mix (Applied Biosystems, Forest City, CA, USA), 2 µL template DNA, and 1.5 µL of species-specific primers (900 nM final concentration) and FAM-labeled MGB probes (100 µM final concentration). All unknown samples were performed in duplicate. DNA extraction and qPCR technique procedures are described elsewhere [39]. 

### 4.5. Participants Data

Participant demographic and household geolocation and characteristics were obtained from CISM DSS. Participants’ HIV status was determined by rapid test; HIV test (Abbott Laboratories, North Chicago, IL, USA) and Uni-Gold HIV test (Trinity Biotech, Bray, Ireland). 

Water and sanitation facilities were defined according to the WHO/UNICEF Joint Monitoring Program [44]. Household water sources were classified as: piped water inside the household, piped water outside the household (in the backyard), fountain, protected well (with a cover and an elevated platform), unprotected well (without a cover and/or an elevated platform), and other water sources. Household sanitation facilities were classified as: toilet connected to a septic tank, improved latrine (latrine with a tank isolating the feces from the environment), unimproved latrine (without a tank that protect the feces from the environment), or without a latrine. 

A socioeconomical wealth index was created to attribute a household socioeconomic score (SES) to each household of Manhiça district. It was based on household characteristics and assets possession collected during CISM DSS. A multiple correspondence analysis (MCA) with 17 characteristics was conducted to determine the weights of every characteristic or asset [45]. We excluded water and sanitation variables to avoid over adjustment. Manhiça district household SES was then divided in tertiles to classify households in poor SES status, middle SES, and rich SES. 

### 4.6. Data Analysis

Participant population and STH and protozoan infections were described using absolute and relative frequency. Categorical variables were expressed as absolute frequency and percentage and they were compared with Fisher’s exact test. 

We estimated the odds ratio for infection with at least one STH infection or infection with at least one enteric protozoon. Although *S. stercoralis* is not formally considered a STH by WHO we included it in the STH group for this analysis. We fitted a logistic regression model. The models were selected using backward procedure. For water source explanatory variable, we adjusted our estimations for age, sex, latrine type, socioeconomical score, and distance to the health post (Euclidean distance); and for sanitation facility, we adjusted our estimations for age, sex, water source type, socioeconomical score, and distance to the health post. Our references were piped water inside the household and toilet connected to a septic tank. 

We performed statistical analysis and data management and visualization using STATA 16 (StataCorp., College Station, TX, USA) and R Statistical Software Version 3.5.3 [46].

### 4.7. Ethics

The study protocol was reviewed and approved by the National Bioethics Committee for Health in Mozambique (reference: IRB00002657) and by Baylor College of Medicine (H-38221). All child adult guardians signed the informed consent in their choice of language (Portuguese or Changana). In case of illiterate adult guardians, study explanation and inform consent process was conducted in the presence of an independent witness and child guardians put a finger print. Infected participants were treated following the national guidelines.

## Figures and Tables

**Table 1:** Participants sex, age, household socioeconomical status and main water and sanitation facility used per recruitment health center and in total.

Characteristics	Manhiça-Sede (%)	Ilha Josina (%)	Total (%)
**Sex**			
Female	109 (47.0)	87 (50.3)	196 (48.4)
Male	123 (53.0)	86 (49.7)	209 (51.6)
**Age**			
2–5 years old	134 (57.8)	77 (44.5)	211 (52.1)
6–10 years old	98 (42.2)	96 (55.5)	194 (47.9)
**Household socioeconomical status**			
Rich	113 (48.7)	26 (15.0)	139 (34.3)
Middle-class	57 (24.6)	45 (26.0)	102 (25.2)
Poor	28 (12.1)	70 (40.5)	98 (24.2)
Missing	34 (14.7)	32 (18.5)	66 (16.3)
**Water source**			
Piped water inside the household	56 (24.1)	4 (2.3)	60 (14.8)
Piped water outside the household	121 (52.2)	7 (4.0)	128 (31.6)
Fountain	17 (7.3)	5 (2.9)	22 (5.4)
Protected well	7 (3.0)	83 (48.0)	90 (22.2)
Unprotected well	8 (3.4)	62 (35.8)	70 (17.3)
Other	8 (3.4)	0 (0.0)	8 (2.0)
Missing	15 (6.5)	12 (6.9)	27 (6.7)
**Sanitation facility**			
Toilet connected to septic tank	48 (20.7)	1 (0.6)	49 (12.1)
Improved latrine	120 (51.7)	135 (78.0)	255 (63.0)
Unimproved latrine	52 (22.4)	10 (5.8)	62 (15.3)
Without latrine	8 (3.4)	17 (9.8)	25 (6.2)
Missing	4 (1.7)	10 (5.8)	14 (3.5)
**HIV infection**			
Positive	15 (6.5)	3 (1.7)	18 (4.4)
Negative	170 (73.3)	215 (124.3)	385 (95.1)
Missing	0 (0.0)	2 (1.2)	2 (0.5)

**Table 2:** Number and percentage (%) of participants with a soil-transmitted helminth (STH) infection, including *Strongyloides stercoralis,* or enteric protozoan infections.

Intestinal Parasite	Total Multi-Parallel qPCR (%)
**Soil-transmitted helminths**	
*Ascaris lumbricoides*	30 (7.4) ^a^
*Trichuris trichiura*	16 (4.0) ^b^
*Necator americanus*	12 (3.0) ^a^
*Ancylostoma duodenale*	2 (0.5) ^a^
*Strongyloides stercoralis*	35 (8.6) ^a^
At least one STH	78 (19.3) ^a^
**Enteric protozoa**	
*Giardia lamblia*	313 (77.3) ^a^
*Entamoeba histolytica*	3 (0.7) ^a^
*Cryptosporidium parvum/hominis*	9 (5.7) ^c^
At least one enteric protozoan	314 (77.5) ^a^

^a^ Total participants analyzed: n = 405; ^b^ total participants analyzed: n = 397; ^c^ total participants analyzed: n = 157.

**Table 3:** Adjusted odds ratio (OR) for being infected by at least one soil-transmitted helminth (STH), including *Strongyloides stercoralis,* or at least one enteric protozoon per water source or sanitation facility used in the household.

	At Least One STH		At Least One Enteric Protozoon	
	Adjusted OR (95% CI)	*p*-value	Adjusted OR (95% CI)	*p*-value
**Water source ^A^**				
Piped water inside the household	1.00	0.088	1.00	0.010
Piped water outside the household	1.09 (0.47–2.54)		1.25 (0.58–2.68)	
Fountain	1.30 (0.35–4.85)		1.30 (0.34–4.94)	
Protected well	0.37 (0.13–1.09)		2.59 (1.01–6.65)	
Unprotected well	0.41 (0.13–1.26)		5.21 (1.56–17.46)	
Other	1.81 (0.34–9.69)		1.81 (0.19–16.97)	
**Sanitation facility ^B^**				
Toilet connected to septic tank	1.00	0.528	1.00	0.835
Improved latrine	0.62 (0.23–1.71)	0.528	0.81 (0.29–2.26)	
Unimproved latrine	0.45 (0.14–1.44)		0.62 (0.21–1.80)	
Without latrine	0.87 (0.18–4.21)		0.86 (0.16–4.76)	

OR: Odds ratio. ^A^ adjusted for age, sex, latrine type, socioeconomical score and distance to the health post. ^B^ adjusted for age, sex, water source type, socioeconomical score and distance to the health post.

## Data Availability

The data underlying this article will be shared on reasonable request to the corresponding author.

## Supplementary Materials

The following are available online at https://www.mdpi.com/article/10.3390/pathogens10070838/s1. Figure S1: Location of Manhiça-sede and Ilha Josina health centers and location of household study participants. Figure S2: Proportion of participants with soil-transmitted helminth infection per (A) gender, (B) age, (C) socioeconomical status (SES), (D) water source type and (E) sanitation facility type. Figure S3: Proportion of participants with enteric protozoa infection per (A) gender, (B) age, (C) socioeconomical status (SES), (D) water source type and (E) sanitation facility type. Table S1: Species-specific primers used in multi-parallel real-time quantitative qPCR selected for each of the 8 parasites.
