# Supplementary material for: Molecular Detection of Soil-Transmitted Helminths and Enteric Protozoa Infection in Children and Its Association with Household Water and Sanitation in Manhiça District, Southern Mozambique
Source: Pathogens. 2021 Jul 3;10(7):838. doi: 10.3390/pathogens10070838 (PMC8308871; doi:10.3390/pathogens10070838)
Supplement: Supplementary file 1 [file pathogens-10-00838-s001.zip › pathogens-1250057-SI.pdf]

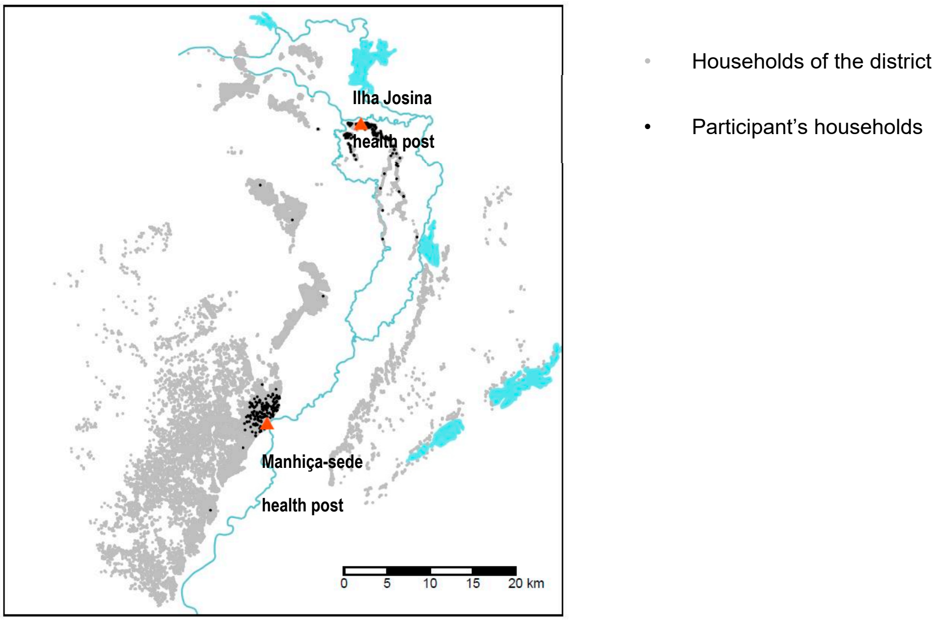

Figure S1. Location of Manhiça-sede and Ilha Josina health centers and location of household study participants. Rivers and lakes are displayed in blue.

Table S1 Species-specific primers used in multi-parallel real-time quantitative qPCR selected for each of the 8 parasites.

Sequence information for the 8 parasites

**Parasite**	**Fwd Primer Sequence**<br>**Rev Primer Sequence**<br>**Probe Sequence (FAM)**	**Target Region**	**Gene Ascension Number**
A. lumbricoides	5'-TGCACATAAGTACTATTTGCGCGTAT-3'<br>5'-CCGCCGACTGCTATTACATCA-3'<br>5'-GAGCCACATAGTAAATT-3'	ITS-1	AB571301.1
C. parvum/hominis	5'-AACTTCACGTGTGTTTGCCAAT-3'<br>5'-CCAATCACAGAATCATCAGAATCG-3'<br>5'-CATATGAAGTTATAGGGATACCAG-3'	DNAJ like protein	XM_625506.1
A. duodenale	5'-GAATGACAGCAAACTCGTTGTTG-3'<br>5'-ATACTAGCCACTGCCGAAACGT-3'<br>5'-ATCGTTTACCGACTTTAG-3'	ITS-2	EU344797.1
N. americanus	5'-CTGTTTGTCGAACGGTACTTGC-3'<br>5'-ATAACAGCGTGCACATGTTGC-3'<br>5'-CTGTACTACGCATTGTATAC-3'	ITS-2	AJ001599.1
S. stercoralis	5'-GAATTCCAAGTAAACGTAAGTCATTAGC-3'<br>5'-TGCCTCTGGATATTGCTCAGTTC-3'<br>5'-ACACACCGGCCGTCGCTGC-3'	18S rRNA	AF279916.2
G. lamblia	5'-CATGCATGCCCGCTCA-3'<br>5'-AGCGGTGTCCGGCTAGC-3'<br>5'-AGGACAACGGTTGCAC-3'	16S rRNA	AJ293299.1
E. histolytica	5'-GTTTGTATTAGTACAAAATGGCCAATTC-3'<br>5'-TCGTGGCATCCTAACTCACTTAGA-3'<br>5'-CAATGAATTGAGAAATGACA-3'	18S rRNA	X75434.1
T. trichiura	5'-TCCGAACGGCGGATCA-3'<br>5'-CTCGAGTGTCACGTCGTCCTT-3'<br>5'-TTGGCTCGTAGGTCGTT-3'	ITS-1	FM991956.1

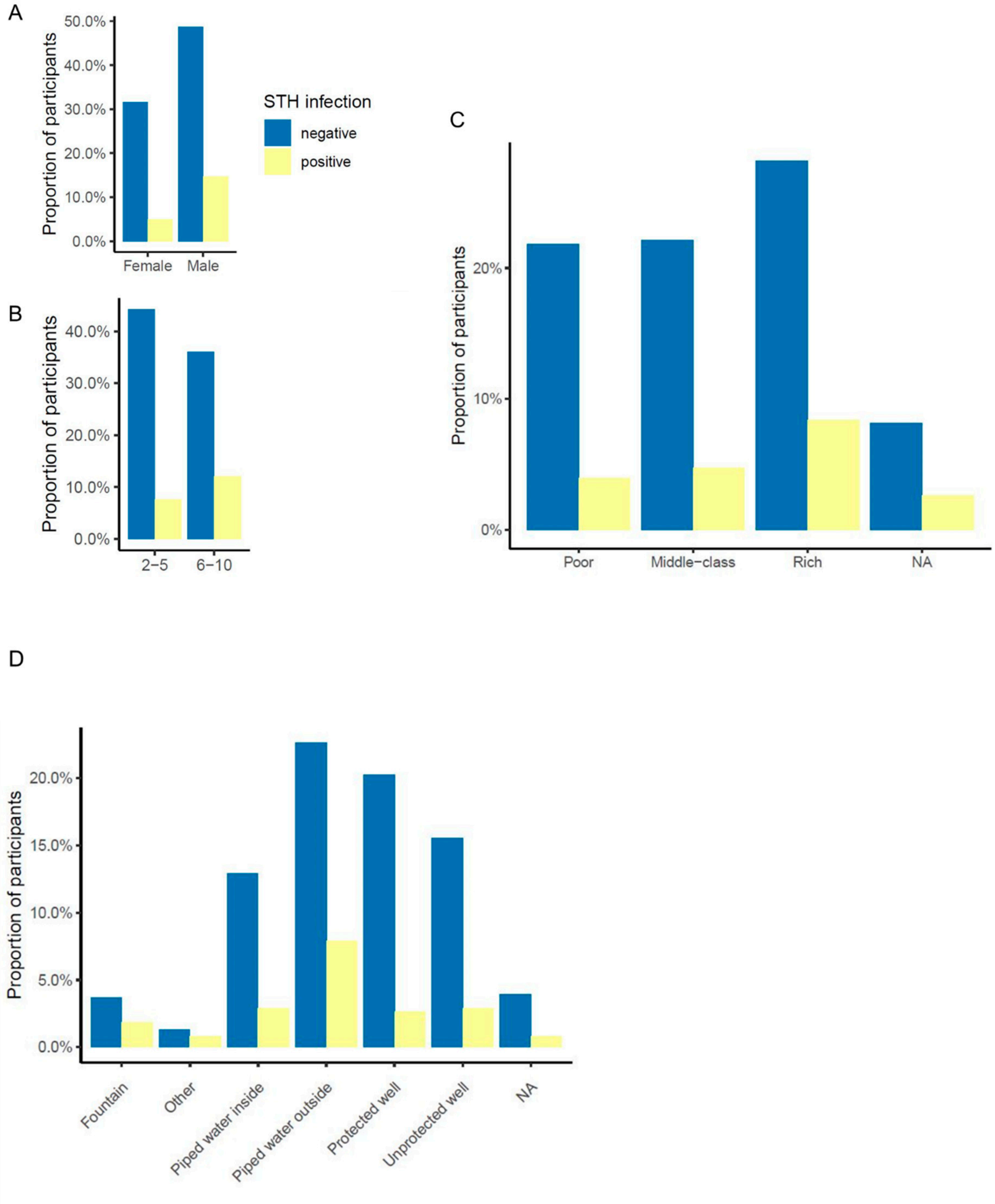
A
Proportion of participants
50.0%
40.0%
30.0%
20.0%
10.0%
0.0%
Female
Male
STH infection
negative
positive
B
Proportion of participants
40.0%
30.0%
20.0%
10.0%
0.0%
2−5
6−10
C
Proportion of participants
20%
10%
0%
Poor
Middle−class
Rich
NA
D
Proportion of participants
20.0%
15.0%
10.0%
5.0%
0.0%
Fountain
Other
Piped water inside
Piped water outside
Protected well
Unprotected well
NA

E

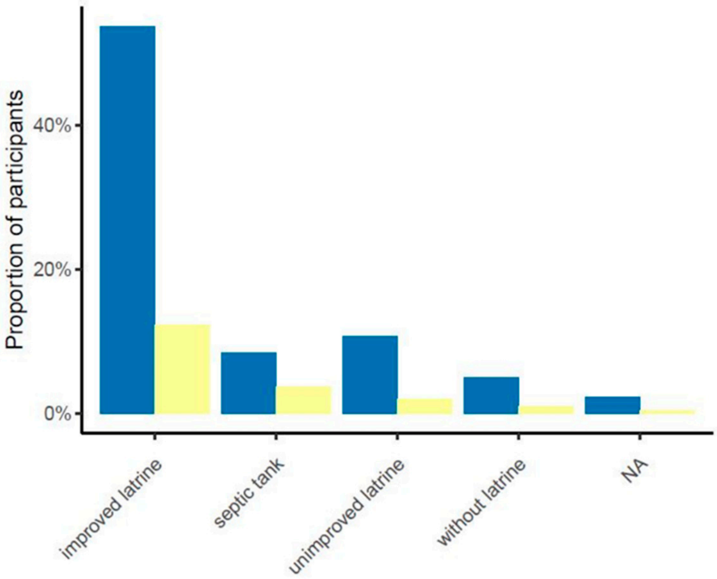

Figure S2. Proportion of participants with soil-transmitted helminth infection per A) gender, B) age, C) socioeconomical status (SES), D) water source type and E) sanitation facility type.

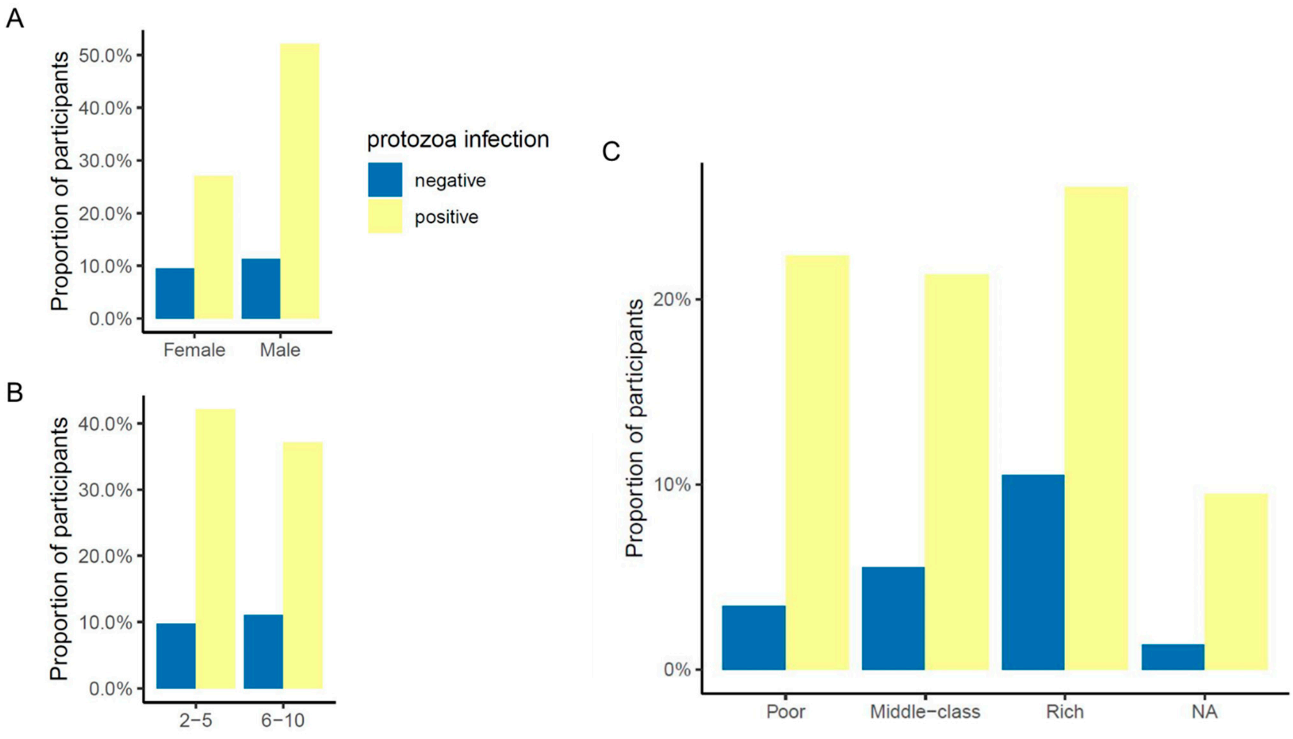
A
Proportion of participants
50.0%
40.0%
30.0%
20.0%
10.0%
0.0%
Female
Male
protozoa infection
negative
positive
B
Proportion of participants
40.0%
30.0%
20.0%
10.0%
0.0%
2−5
6−10
C
Proportion of participants
20%
10%
0%
Poor
Middle−class
Rich
NA

D

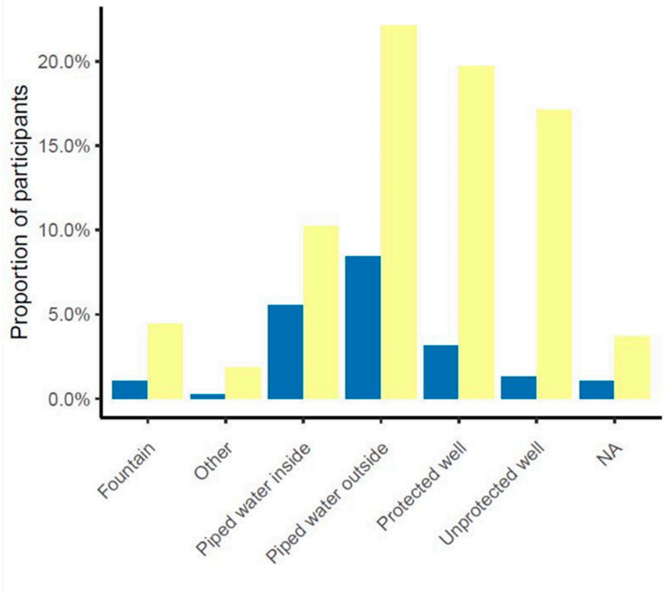

E

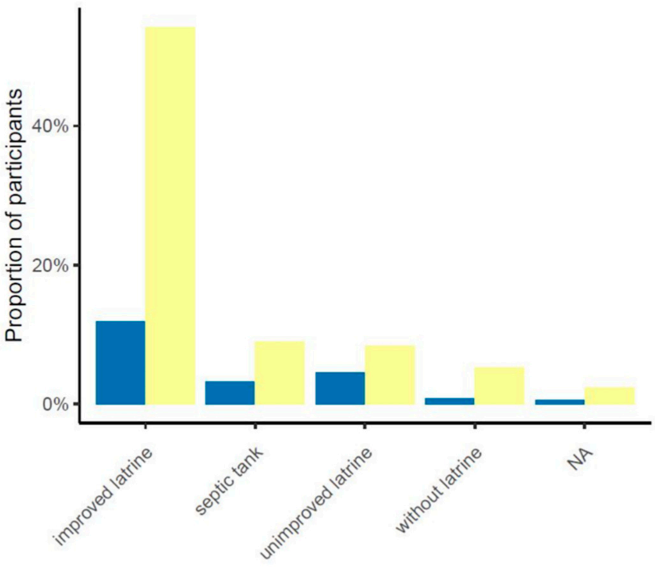

Figure S3. Proportion of participants with enteric protozoa infection per A) gender, B) age, C) socioeconomical status (SES), D) water source type and E) sanitation facility type.
